# Thymalfasin, a promising adjuvant therapy in small hepatocellular carcinoma after liver resection

**DOI:** 10.1097/MD.0000000000006606

**Published:** 2017-04-21

**Authors:** Chao He, Wei Peng, Chuan Li, Tian-Fu Wen

**Affiliations:** Department of Liver Surgery and Liver Transplantation Center, West China Hospital of Sichuan University, Guoxuexiang, Chengdu, Sichuan Province, China.

**Keywords:** hepatocellular carcinoma, liver resection, Milan criteria, prognostic factor, thymalfasin

## Abstract

There is limited information available concerning the effect of thymalfasin (Tα1) as an adjuvant therapy in hepatocellular carcinoma (HCC) patient who received liver resection. The present study aimed to evaluate whether Tα1 can improve the prognosis of small HCC patients after liver resection.

A total of 206 patients with small HCC who underwent liver resection were analyzed in our retrospective cohort study. Patients were divided into 2 groups: group A (resection + Tα1, n = 44) and group B (resection, n = 162). Clinical data, overall survival (OS), and recurrence-free survival (RFS) were compared. Prognostic factors were identified using multivariate analysis.

After a median follow-up of 47.0 months, 134 patients (65%) had recurrence, and 62 patients (30.09%) died. The 1, 3, and 5-year OS rate of patients in group A was 97.7%, 90.6%, and 82.9%, respectively, and 95.1%, 80.5%, and 62.9%, respectively, for patients in group B (*P* = .014). The 1, 3, and 5-year RFS rate of patients in group A was 70.5%, 56.8%, and 53.3%, respectively, and 65.8%, 41.3%, and 32.1%, respectively, for patients in group B (*P* = .015). Multivariate analysis indicated that Tα1 was an independent prognostic factor for both OS (*P* = .015, hazard ratio 0.349, 95% confidence interval 0.149–0.816) and RFS (*P* = .019, hazard ratio 0.564, 95% confidence interval 0.349–0.910).

Tα1 as an adjuvant therapy after liver resection may improve the prognosis of small HCC patients after liver resection.

## 1 Introduction

Hepatocellular carcinoma (HCC) is 5th common malignancies and the 3rd-leading cause of cancer-related death worldwide. Because of high hepatitis B virus (HBV) infection, China alone accounts for about 50% of the total number of cases and deaths.^[1,2]^ Although surgical techniques and perioperative management have improved, survival remains very poor in HCC patients. Therefore, continuing efforts to explore new approaches to impede recurrence and improve the outcome of HCC is necessary. Immunotherapy has been shown to have potential benefit but evidence is not strong enough.^[3,4]^ Thymalfasin (thymosin α1, Tα1, commercial name: ZADAXIN) is a naturally occurring thymic polypeptide of 28 amino acids.^[5]^ The mechanism of this drug is related to its immune-modulating activities, and Tα1 has been widely used in various diseases, including some infection disease and malignances.^[6]^ Previous investigations have shown that Tα1 may improve the outcomes of HCC patients who underwent transarterial chemoembolization (TACE).^[7,8]^ However, whether Tα1 can improve the prognosis of small HCC patients who received liver resection has not been confirmed and the underlying mechanisms remains unclear. The present study was designed to evaluate the efficacy of Tα1 as an adjuvant therapy in patients with small HCC who underwent liver resection.

## 2 Methods

### 2.1 Patients

**Figure 1:**
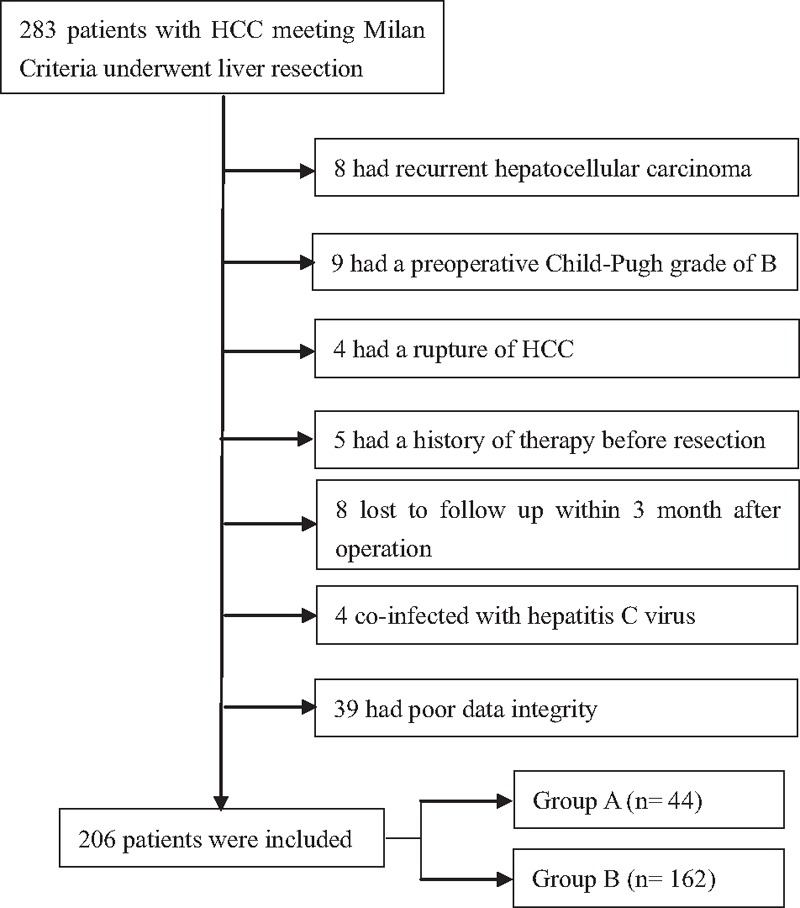
Flowchart of the process for patients’ selection.

This study was approved by the Ethics Committee of West China Hospital, Sichuan University. The HCC patients who underwent liver resection in the Department of Liver Surgery and Liver Transplantation Center of West China Hospital between February 2007 and February 2013 were identified from our prospectively maintained database. During the period, 283 small HCC patients (within Milan criteria) received curative resection in our department. Diagnoses of HCC were confirmed by postoperative histopathologic examination. Microvascular invasion (MVI) was identified under a light microscope by pathologists. HCC was histologically classified using the Edmondson–Steiner classification. Clinical data, follow-up data, recurrence, and survival data were retrieved from our prospectively maintained database. In our study, inclusion criteria were as follows: primary HBV-related small HCC (solitary tumor <5 cm in diameter or ≤3 nodules, each of them <3 cm in diameter, without major vascular invasion or distant metastasis); receiving liver resection as initial therapy in our hospital; and liver reserve function Child–Pugh grade A. Exclusion criteria included the following: extra-hepatic malignancies; previous resection, TACE, ablative therapies, or liver transplantation; loss to follow-up within 3 months after liver resection; poor liver reserve function with a Child–Pugh grade B or C; rupture of HCC; coinfection with hepatitis C virus; simultaneous splenectomy; and previously treated with Tα1. Based on our inclusion and exclusion criteria, 77 patients were excluded. Excluded patients were as follows: 8 had recurrent HCC, 9 had a preoperative Child–Pugh grade of B, 4 had a rupture of HCC, 5 had a history of therapy before resection, 8 lost to follow-up within 3 months after operation, 4 coinfected with hepatitis C virus, and 39 had poor data integrity. Finally, a total of 206 patients were included in the analysis. Patients were divided into 2 groups based on whether they received Tα1 adjuvant therapy: group A (liver resection plus Tα1, n = 44), group b (liver resection only, n = 162). Details about patient selection are shown in Fig. 1. Current regimen of Tα1 (ZADAXIN): 1.6 mg subcutaneously twice per week for at least 26 weeks.

### 2.2 Follow-up visit

All the 206 patients were regularly followed up at the 1st, 3rd, and 6th months in the 1st half year after surgery, every 3 months during the following 3 years, and every 6 months in the subsequent years. Antiviral therapy was administered to patients with positive hepatitis B DNA before and after operation. Physical examination, blood cell and differential counts, alpha fetoprotein level, liver function test and HBV-DNA, and radiology examination were included in the follow-up examinations. Tumor recurrence was diagnosed according to positive imaging findings and arising of tumor marker (alpha fetoprotein) or pathologic report of biopsy or resection. Once identified, patients were advised to receive additional treatment. For example, liver transplantation, resection, ablative therapy, TACE, or sorafenib. The last follow-up date was the end of June 2016.

### 2.3 Statistical analysis

We used SPSS software version 20.0 (SPSS Company, Chicago, IL) for windows to perform statistical analysis. The continuous variables are expressed as the mean ± the standard deviation. The categorical variables are presented as numbers (percentages). Categorical data were compared by the chi-square test or Fisher exact test. Continuous variables were compared by independent *t* test for normal distributed data or Mann–Whiney *U* test for skewness-distributed data. The overall survival (OS) and recurrence-free survival (RFS) were analyzed by the Kaplan–Meier method, and the differences were analyzed by a log-rank test. Multivariate Cox proportional hazards regression analysis was used to evaluate the prognostic factors. Calculated *P* values were 2-sided, and a *P* value <.05 was considered statistically significant.

Neutrophil to lymphocyte ratio (NLR) was defined as absolute neutrophil counts divided by lymphocyte counts. For group A, NLR0 was defined as NLR in the first follow-up visit. AS for group B, NLR0 was defined as NLR before administration of Tα1. dNLR1, dNLR2, and dNLR3 were defined as the NLR of 2nd, 3rd, and 4th follow-up visit minus NLR0. Data of NLR were excluded if there was clinical symptoms or signs of sepsis at the time of blood sampling for NLR, or white blood cell counts >10^109/L.

## 3 Results

### 3.1 Baseline characteristics

In the present research, a total of 206 patients were included in the analysis. As described in Table 1. There were 178 males (86.4%) and 28 females (14.6%), with the mean age of 50.2 ± 11.8 (range from 21 to 78) year. The median follow-up time 47.0 months (range from 21.00 to 78.00). A total of 174 patients (84.5%) had 1 nodule, and 32 (15.5%) patients had 2 or 3 nodules; 78 patients (37.9%) had a nodule <3 cm in diameter, and 128 (62.1%) had a nodule 3 to 5 cm in diameter. A total of 178 (86.4%) patients had cirrhosis, 108 (52.4%) patients had HBV-DNA levels higher than 10^3 IU/L. The baseline characteristics of patients in the 2 groups are described in Table 1. There was no significant difference between the 2 groups in the baseline characteristics and the number of patients who received adjuvant therapy after recurrence (Table 2). In group A, all of the 44 patients were administrated Tα1 with a median treatment time of 33.9 months (range from 6 to 93 months).

**Table 1:**
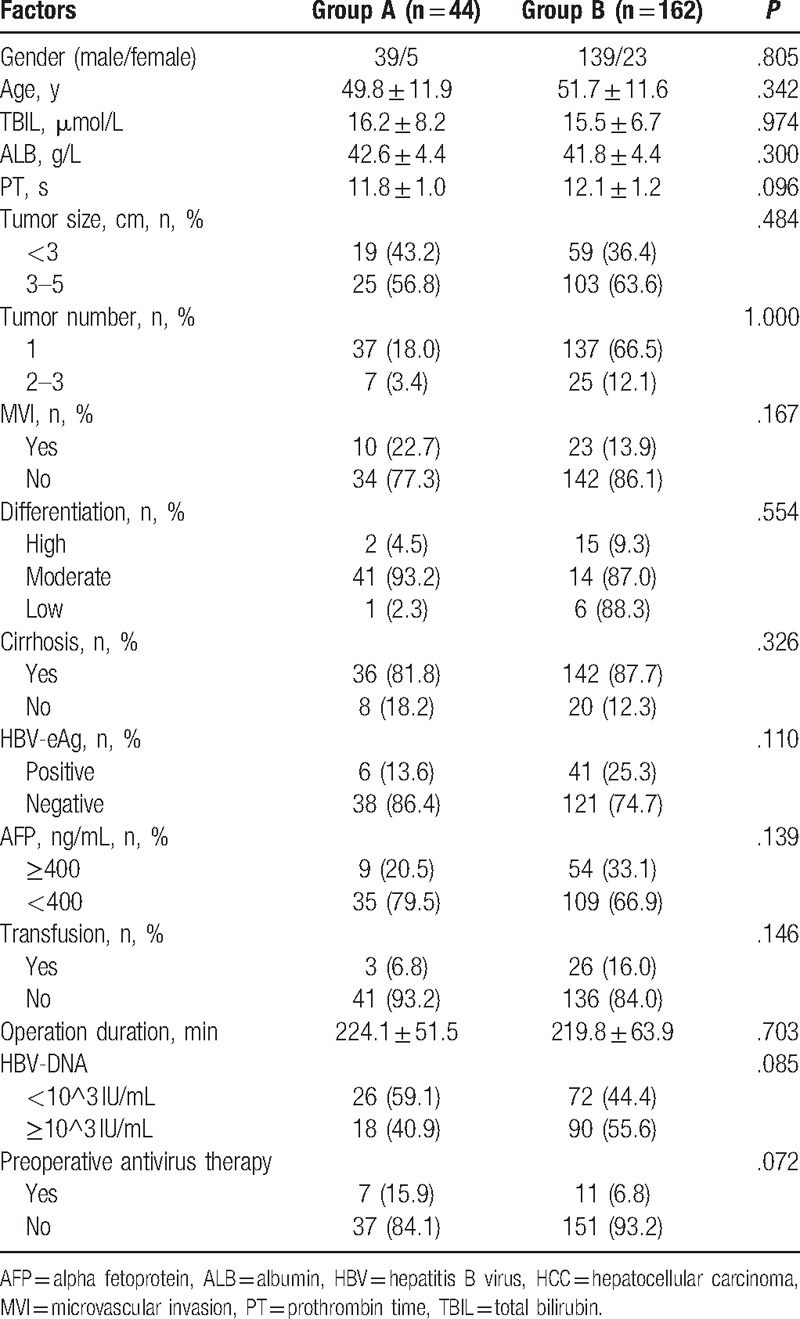
Demographic and clinical data of 206 small HCC patients according to thymalfasin.

**Table 2:**
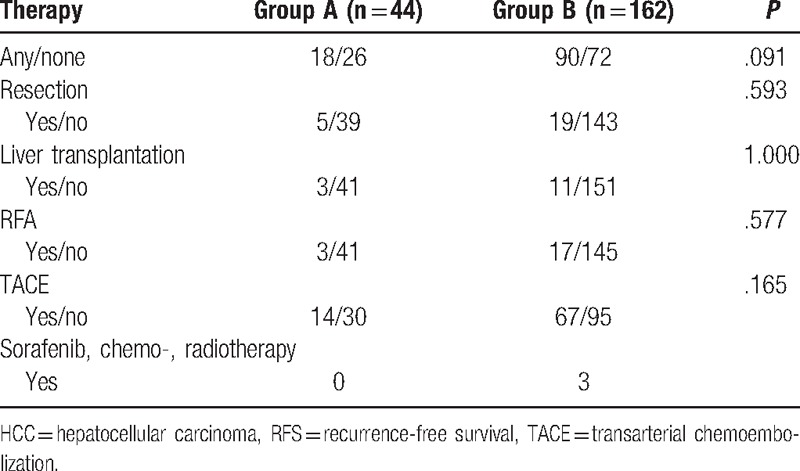
Adjuvant therapy of 206 small HCC patients according to thymalfasin after liver resection.

### 3.2 Impact of Tα1 on OS

**Figure 2:**
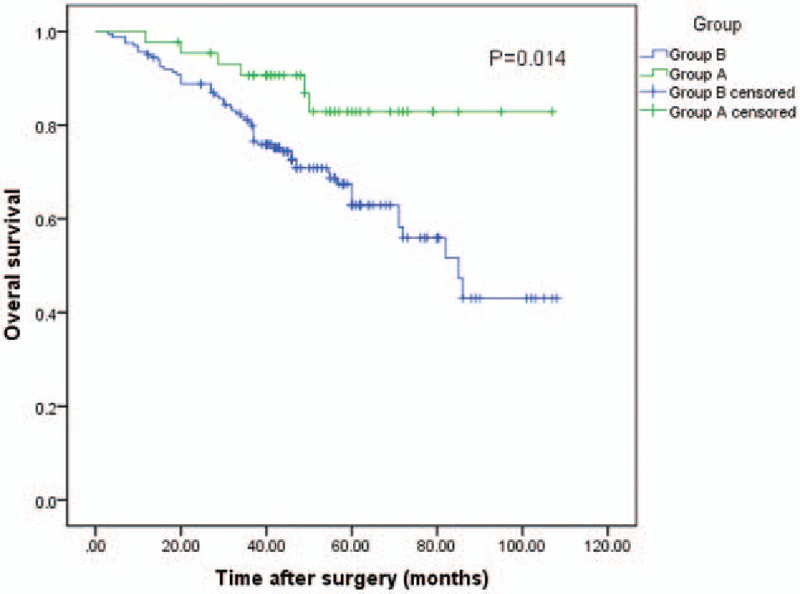
The graph shows the OS curve of group A (liver resection plus Tα1, n = 44) and group B (liver resection, n = 162). Group A had better OS than group B (log-rank test, *P* = .014). OS = overall survival, Tα1 = thymalfasin.

After a median follow-up of 47.0 months, 134 patients (65%) had recurrence, and 62 patients (30.1%) died. The 1, 3, and 5-year OS rate of all 206 patients was 95.6, 82.7, and 67.0%, respectively. When dividing the patients into 2 groups by Tα1, the 1, 3, and 5-year OS rate of patients in group A was 97.7, 90.6, and 82.9%, respectively, and 95.1, 80.5, and 62.9%, respectively, for patients in group B. The median OS time was 94.8 months (95%CI 85.7–103.8) for group A, 74.9 months (95%CI 68.2–81.7) for group B (log-rank test, *P* = .014, Fig. 2).

### 3.3 Impact of Tα1 on RFS

**Figure 3:**
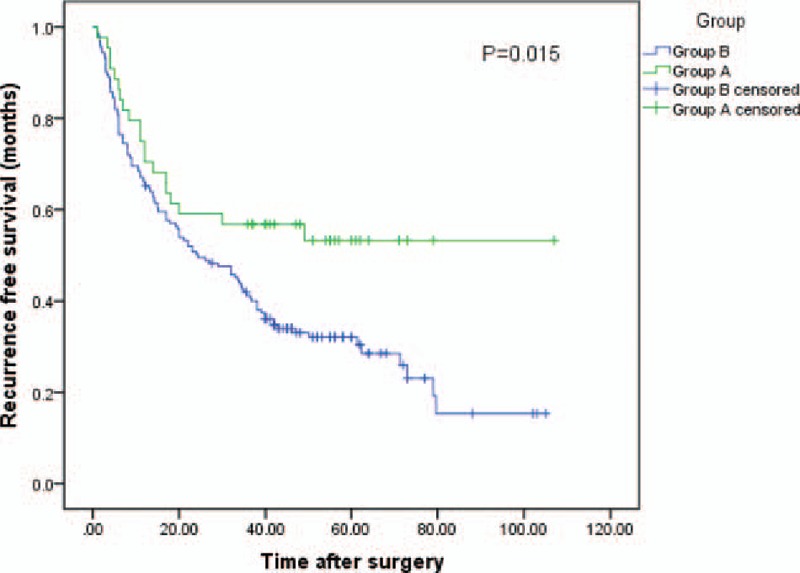
The graph shows the RFS curve of group A (liver resection plus Tα1, n = 44) and group B (liver resection, n = 162). Group A had better RFS than group B (log-rank test, *P* = .015). RFS = recurrence-free survival, Tα1 = thymalfasin.

The 1, 3, and 5-year RFS rate of all 206 patients was 67.8%, 44.7%, and 36.5%, respectively. When dividing the patients into 2 groups by Tα1, the 1, 3, and 5-year RFS rate of patients in group A was 70.5%, 56.8%, and 53.3%, respectively, and 65.8%, 41.3%, and 32.1%, respectively, for patients in group B (*P* = .015). The median RFS time was 63.4 months (95%CI 49.3–77.6) for group A, 39.6 months (95%CI 33.3–46.0) for group B (log-rank test, *P* = .015 Fig. 3).

### 3.4 Impact of Tα1 on dynamic NLR change

Because of lacking the integrity of the follow-up blood cell data or patients having clinical symptoms or signs of sepsis at the time of blood sampling for NLR, patient number has changed in the analysis of dynamic NLR change. In group A, dNLR1 increased in 4 patients (3.5%) and decreased in 31 patients (64.6%), in group B, 109 patients (96.5%) increased and 17 patients (35.4%) decreased. dNLR3 increased in 4 (4.3%) patients, decreased in 31 (68.9%) patients in group A, increased in 10 (96.5%), decreased in 17patients (35.4%) in group B. dNLR6 increased in 2 patients (2.7%), decreased in 30 patients (66.7%) in group A, increased in 73 patients (97.3%), decreased in 15 patients (33.3%) in group B. *P* value of dNLR1, dNLR3, and dNLR6 are all <.001, as shown in Table 3.

**Table 3:**
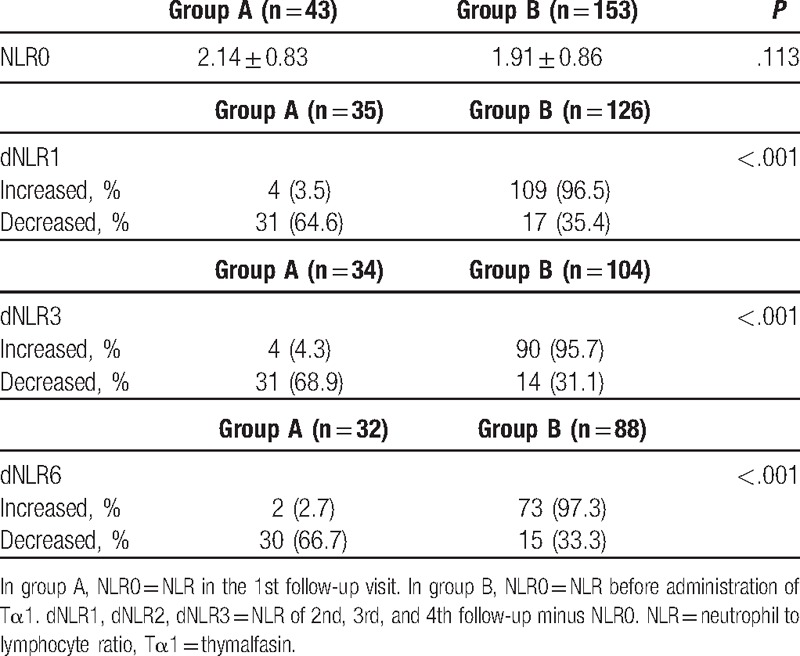
Dynamic NLR changes.

### 3.5 Univariate and multivariate analysis

To identify the prognostic factors for OS and RFS of small HCC after liver resection, 17 potential variables were analyzed. As shown in Table 4, univariate analysis suggested that MVI, transfusion, total bilirubin, and Tα1 were significantly related to OS. Multivariate analysis demonstrated that MVI (*P* = .001, hazard ratio [HR] = 2.704, 95% confidence interval [CI] 1.510–4.842) and transfusion (*P* = .009, HR = 2.179, 95% CI 1.215–3.907), and Tα1 (*P* = .015, HR = .349, 95% CI 0.149–0.816) were the prognostic factors for OS of small HCC patients after liver resection. As shown in Table 5, univariate analysis suggested that MVI, tumor number, cirrhosis, HBV-DNA, and Tα1 were significantly related to the RFS. Multivariate analysis demonstrated that microvascular (*P* = <.001, HR = 2.481, 95%CI 1.615–3.812), tumor number (*P* = .001, HR = 2.113, 95% CI 1.380–3.234), cirrhosis (*P* = .023, HR = 1.966, 95%CI 1.100–3.515), and Tα1 (*P* = .019, HR = .564, 95%CI .349–.910) were independent prognostic factors for RFS of small HCC patients.

**Table 4:**
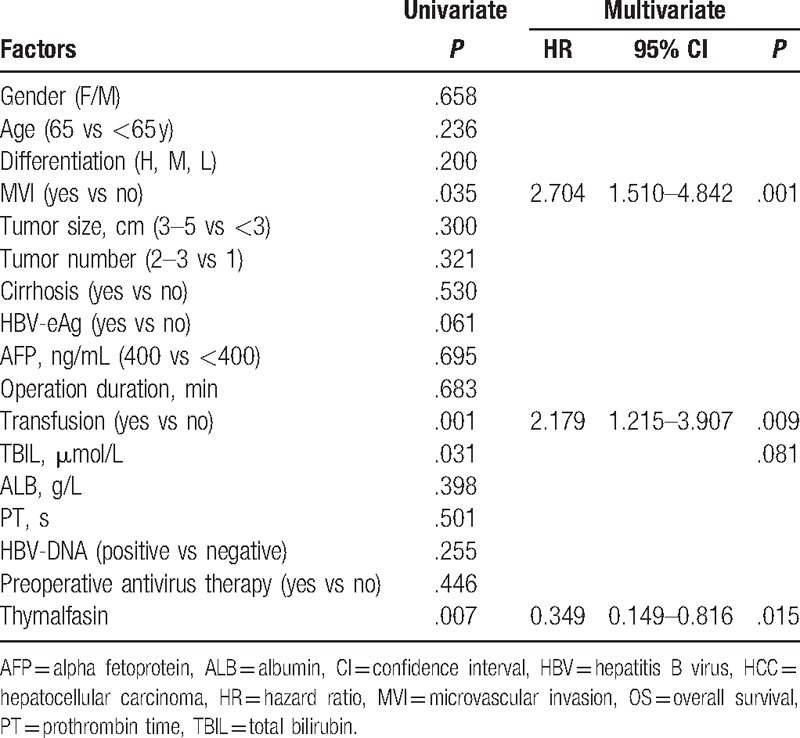
Univariate and multivariate analyses of prognostic factors for OS of 206 small HCC patients after liver resection.

**Table 5:**
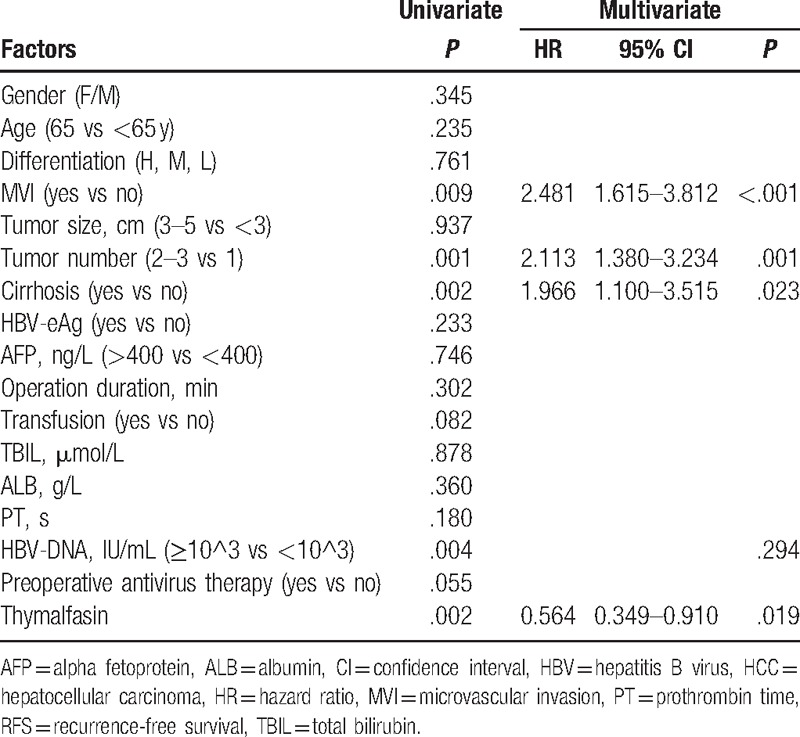
Univariate and multivariate analyses of prognostic factors for RFS of 206 small HCC patients after liver resection.

## 4 Discussion

Liver resection has been widely accepted as standard treatment for HCC patients. However, recurrence rate is high as 70% at 5 years.^[3]^ Risk factors for recurrence are multiple and prevalent. Consistent with previous research, our research confirmed MVI, tumor number, and cirrhosis were independent risk factors for small HCC recurrence. In small HCC, about 15% to 35% cases are with MVI,^[9]^ about 60% to 90% cases are with cirrhosis,^[10,11]^ and about 15% to 45% cases are multifocal.^[10,12,13]^ Therefore, even in small HCC, 5-year RFS rate is only about 40%,^[14,15]^ improvements in preventing recurrence and prolonging OS are essential. However, methods to prevent recurrence are limited. Adjuvant therapy, such as TACE and sorafenib, although are generally used for HCC patients who have risk factors of recurrence, remains controversial.^[8,16]^ TACE is restricted by invasive nature, possibility of severe complications. As for sorafenib, its application is mainly compromised by high cost which is not affordable for most HCC patients. Immunotherapy has shown some potential efficacy, but evidence is not strong enough.^[3,17]^ Previous investigations have shown Tα1 may improve the outcome of unresectable HCC patients who received TACE.^[7,18,19]^ However, little is known about its efficacy in HCC patients who received liver resection. Shuqun et al^[20]^ report that Tα1 plus lamivudine postoperatively compared to lamivudine alone may suppress HBV reaction, delay the recurrent time, and prolong the survival for HCC patients. However, their sample size was small and without stratification based on BCLC staging system. Our research is the first one to focus the efficacy of Tα1 in small HCC patients who underwent liver resection. The present study demonstrated that Tα1 adjuvant therapy resulted in significant better OS and RFS than resection only. Several mechanisms may be related to the efficacy of Tα1 in improving the prognosis of HCC patients.

In recent years, accumulated evidence suggest that the systemic inflammatory response plays a significant role in various malignancies including HCC. NLR is one of the systemic inflammation markers, high postoperative NLR has been shown to be related to poor prognosis of HCC, and reduction of postoperative NLR is associated better prognosis.^[15]^ Our research first reports that Tα1 can reduce postoperative NLR. The decline of NLR was associated with reduction of neutrophils and increment of lymphocytes. As lymphocytes have pivotal roles in inhibiting proliferation and metastatic activity of tumor cells. A relative lymphopenia may reflect deficient immune response to malignances. On the other hand, increased neutrophils can increase the level of circulating vascular endothelial growth factor, angiopoietin-1, and matrix metalloproteinase-9 which are major contributors to tumor-related angiogenesis.^[21,22]^ Second, the immune response against tumor is weakened in HCC patients.^[23,24]^ Tα1 can stimulate both adaptive and innate immune system against tumor by various paths. For instance, augmenting T-cell function, modulating cytokine and chemokine production, and augmenting the function of macrophages and other immune cells involving innate immune system.^[6,25]^ Furthermore, Tα1 even suppress proliferation and induce apoptosis of tumor cells.^[26]^ Third, postoperative active HBV replication has been proved to be associated with early HCC recurrence and shortened OS. Several studies have proved that combination of antivirus therapy and Tα1 postoperatively may be more effective in control HBV infection, and thus improve the prognosis of HCC. Therefore, Tα1 may improve the prognosis of HBV-related HCC by inhibiting HBV active replication.^[20,27]^

There are several limitations in our study. First, this is a retrospective analysis from our single institution, and the sample size is relatively small. Second, the analysis of the dynamic change of NLR did not incorporate all the recruited patients, because of lacking the integrity of the follow-up blood cell data and patients had clinical symptoms or signs of sepsis at the time of blood sampling for NLR. Therefore, the result of dynamic NLR change must be tested in prospective research. Third, the present study only investigates the effect of Tα1 in small HCC patients who underwent liver resection, it is uncertain its effect in other stages of HCC, or other modalities of treatment.

In conclusion, our study demonstrated that Tα1 as adjuvant therapy in small HCC after liver resection may delay recurrence and prolong OS. It is rational for small HCC patients who have high risk for recurrence after resection to receive Tα1 adjuvant therapy.

## Acknowledgments

The authors thank Scientific and Technological Support Project of Sichuan Province (2015SZ0049) for the support.

## References

[1] Torre LA Bray F Siegel RL Global cancer statistics, 2012. CA Cancer J Clin 2015;65:87–108. 25651787 10.3322/caac.21262

[2] Ferlay J Shin HR Bray F Estimates of worldwide burden of cancer in 2008: GLOBOCAN 2008. Int J Cancer 2010;127:2893–917. 21351269 10.1002/ijc.25516

[3] Forner A Llovet JM Bruix J Hepatocellular carcinoma. Lancet 2012;379:1245–55. 22353262 10.1016/S0140-6736(11)61347-0 PMC13537732

[4] Jeng WJ Lin CC Chen WT Adjuvant therapy for hepatocellular carcinoma after curative treatment. Dig Dis 2014;32:747–54. 25376293 10.1159/000368017

[5] Goldstein AL Guha A Zatz MM Purification and biological activity of thymosin, a hormone of the thymus gland. Proc Nat Acad Sci USA 1972;69:1800–3. 4505657 10.1073/pnas.69.7.1800 PMC426805

[6] Moretti S Oikonomou V Garaci E Thymosin alpha1: burying secrets in the thymus. Expert Opin Biol Ther 2015;15 Suppl 1:S51–8. 26098878 10.1517/14712598.2015.1044895

[7] Gish RG Gordon SC Nelson D A randomized controlled trial of thymalfasin plus transarterial chemoembolization for unresectable hepatocellular carcinoma. Hepatol Int 2009;3:480–9. 19669251 10.1007/s12072-009-9132-3 PMC2748379

[8] Zhong JH Li LQ Postoperative adjuvant transarterial chemoembolization for participants with hepatocellular carcinoma: a meta-analysis. Hepatol Res 2010;40:943–53. 20887328 10.1111/j.1872-034X.2010.00710.x

[9] Ko CJ Chien SY Chou CT Factors affecting the prognosis of small hepatocellularcarcinoma in Taiwanese patients following hepatic resection. Gastroenterology 2011;25:485–91. 10.1155/2011/790528 PMC3202355 21912759

[10] Hosaka T Ikeda K Kobayashi M Predictive factors of advanced recurrence after curative resection of small hepatocellular carcinoma. Liver Int 2009;29:736–42. 19018978 10.1111/j.1478-3231.2008.01901.x

[11] Hong YJ Kim SH Choi GH Long-term outcome after liver resection and clinicopathological features in patients with small hepatocellular carcinoma. Korean J Hepatobiliary Pancreat Surg 2011;15:199–205. 26421040 10.14701/kjhbps.2011.15.4.199 PMC4582469

[12] Baccarani U Benzoni E Adani GL Superiority of transplantation versus resection for the treatment of small hepatocellular carcinoma. Transplant Proc 2007;39:1898–900. 17692647 10.1016/j.transproceed.2007.05.045

[13] Shimozawa N Hanazaki K Longterm prognosis after hepatic resection for small hepatocellular carcinoma. J Am Coll Surg 2004;198:356–65. 14992736 10.1016/j.jamcollsurg.2003.10.017

[14] Zhu YB Xu X Zheng SS Association of microvascular invasion with recurrence and prognosis of patients with small hepatocellular carcinoma undergoing liver transplantation. Zhejiang Da Xue Xue Bao Yi Xue Ban 2014;43:658–63. 25644564 10.3785/j.issn.1008-9292.2014.11.004

[15] Peng W Li C Wen TF Neutrophil to lymphocyte ratio changes predict small hepatocellular carcinoma survival. J Surg Res 2014;192:402–8. 24998425 10.1016/j.jss.2014.05.078

[16] Zhong J-H Du XK Xiang BD Adjuvant sorafenib in hepatocellular carcinoma: a cautionary comment of STORM trial. World J Hepatol 2016;8:957–60. 27621761 10.4254/wjh.v8.i23.957 PMC4990759

[17] Yang S Lin Q Lin W Effect of adjuvant interferon therapy on hepatitis B virus-related hepatocellular carcinoma: a systematic review. World J Surg Oncol 2016;14:159. 27282382 10.1186/s12957-016-0912-7 PMC4899889

[18] Stefanini GF Foschi FG Castelli E Alpha-1-thymosin and transcatheter arterial chemoembolization in hepatocellar carcinoma patients: a preliminary experience. Hepatogastroenterology 1998;45:209–15. 9496515

[19] Cheng SQ Wu MC Chen H Transcatheter hepatic arterial chemoembolization and thymosin alpha1 in postoperative treatment of hepatocellular carcinoma. Zhonghua Zhong Liu Za Zhi 2004;26:305–7. 15312371

[20] Shuqun C Mengchao W Han C Antiviral therapy using lamivudine and thymosin alpha1 for hepatocellular carcinoma coexisting with chronic hepatitis B infection. Hepatogastroenterology 2006;53:249–52. 16608033

[21] Kuang DM Zhao Q Wu Y Peritumoral neutrophils link inflammatory response to disease progression by fostering angiogenesis in hepatocellular carcinoma. J Hepatol 2011;54:948–55. 21145847 10.1016/j.jhep.2010.08.041

[22] Kusumanto YH Dam WA Hospers GA Platelets and granulocytes, in particular the neutrophils, form important compartments for circulating vascular endothelial growth factor. Angiogenesis 2003;6:283–7. 15166496 10.1023/B:AGEN.0000029415.62384.ba

[23] Meng W Bai B Bai Z The immunosuppression role of alpha-fetoprotein in human hepatocellular carcinoma. Discov Med 2016;21:489–94. 27448785

[24] Zhao F Korangy F Greten TF Cellular immune suppressor mechanisms in patients with hepatocellular carcinoma. Dig Dis 2012;30:477–82. 23108303 10.1159/000341695 PMC3530892

[25] Serafino A Pierimarchi P Pica F Thymosin alpha1 as a stimulatory agent of innate cell-mediated immune response. Ann N Y Acad Sci 2012;1270:13–20. 23050812 10.1111/j.1749-6632.2012.06707.x

[26] Fan YZ Chang H Yu Y Thymosin alpha1 suppresses proliferation and induces apoptosis in human leukemia cell lines. Peptides 2006;27:2165–73. 16644063 10.1016/j.peptides.2006.03.012

[27] Kim BK Park JY Kim DY Persistent hepatitis B viral replication affects recurrence of hepatocellular carcinoma after curative resection. Liver Int 2008;28:393–401. 18028321 10.1111/j.1478-3231.2007.01625.x

